# The Role of Inflammatory Cytokines in the Pathogenesis of and Protection Against *Helicobacter pylori*: Comment on "Altered cytokine production in patients with Helicobacter pylori infection"

**DOI:** 10.34172/mejdd.2025.422

**Published:** 2025-04-30

**Authors:** Zohreh Jadali

**Affiliations:** ^1^Department of Immunology, School of Public Health, Tehran University of Medical Sciences, Tehran, Iran

 I read the article by Safikhani Mahmoodzadeh et al, which examined serum levels of some inflammatory cytokines inindividuals infected with *Helicobacter pylori*.They found a significant difference in the levels of IL-2, IL-4, IL-17A, IL-17F, IL-22, TNF-α, and IFN-γ between patients and controls.^1^

 These findings are important because non-physiological and uncontrolled inflammatory processes are hallmarks of *H. pylori* infection. Therefore, measuring cytokine profiles as inflammatory biomarkers may be validated for clinical use. Nonetheless, the results of cytokine assays are difficult to interpretbecause their role may differ depending on the cellular source, target, and phase of the immune response. For instance, all the above cytokines can have both pro- and anti-inflammatory activities depending on context.

*Helicobacter pylori* infection promotes multiple immune response activities such as the expansion of proinflammatory T cells, including Th1 (the primary source of IL-2, IFN-γ, TNF-*α*) and IL-17/IL-22 producing Th17 cells that can produce a robust antimicrobial inflammatory response.^2^ However, over-induction of Th1 and Th17 cells appears to be the culprit for persistent inflammation in gastric cancer and *H*. *pylori *infection. Functional plasticity of T cell subsets (like Th17 cells) could also have been an influencing factor. Inflammatory milieu, allowing Th17 cells to acquire IFN-γ- or IL-4-producing capacity. Research has shown that the expansion of IFN-γ producing Th17 cells in disease-susceptible hosts contributes to intestinal pathology.^3^ Another important point is the existence of different IL-17 isoforms (6 structurally related cytokines) that have different and sometimes even opposing functions. Among them, IL-17A and IL-17F have a broad-ranging influence on inflammatory responses. Th17 responses, especially IL-17A, have been shown to play a key role in the inflammatory response during colonization of* H*. *pylori* in gastric mucosa. Several studies conclude that increased production of IL-17A correlates with severe disease and poor prognosis.^4^

 Synergistic (IL-17 and IL-22), antagonistic (IFN-γ and IL-4), and additive (IL-17 and TNF-α) interactions between these cytokines can also add to the complexity of accurate data interpretation.

 The increased level of IL-4 (a Th2 cell signature cytokine) can be discussed from this perspective.IL-4 can blockTh1-polarizing/inflammatory cytokine gene expression. This increase may have two opposing effects on inflammatory responses. IL-4 overproduction may act as a compensatory mechanism for controlling the balance of pro- and anti-inflammatory cytokines. Alternatively, aberrant production of IL-4 may play a contributory pathogenic role in chronic infection.^5^

 The role of cytokine receptors is another point that should not be forgotten in this discussion.

 Cytokines generally play a biological *role* by binding to the corresponding cytokine receptors on the cell surface. Therefore, knowledge of cytokine–receptor interaction is a gateway for defining disease processes. For instance, the binding of IL-17 to its receptors has a key role in the regulation of inflammation during *H. pylori* infection. Some research indicates that loss of the IL-17 receptor is required to limit the extent of immunopathology following *H. pylori *infection.^4^

 Overall, dysregulation of pro-/anti-inflammatory cytokines plays an important role in the pathophysiology of *H. pylori*. Therefore, further research studies are warranted to understand the mechanisms and pathways underlying cytokine imbalance in *H. pylori.*
